# Mid-Regional Proadrenomedullin and Mid-Regional Proatrial Natriuretic Peptide Clearance Predicts Poor Outcomes Better Than Single Baseline Measurements in Critically Ill Patients With Pneumonia: A Retrospective Cohort Study

**DOI:** 10.7759/cureus.15285

**Published:** 2021-05-28

**Authors:** Jos Van Oers, Johannes Krabbe, Evelien Kemna, Yvette Kluiters, Piet Vos, Dylan De Lange, Armand Girbes, Albertus Beishuizen

**Affiliations:** 1 Department of Intensive Care Medicine, Elisabeth-TweeSteden Ziekenhuis, Tilburg, NLD; 2 Department of Clinical Chemistry, Medisch Spectrum Twente, Enschede, NLD; 3 Department of Clinical Chemistry, Elisabeth-TweeSteden Ziekenhuis, Tilburg, NLD; 4 Department of Intensive Care Medicine, University Medical Centre Utrecht, University Utrecht, Utrecht, NLD; 5 Department of Intensive Care Medicine, Amsterdam University Medical Center, Medical Centres, VU University Medical Centre, Amsterdam, NLD; 6 Department of Intensive Care Medicine, Medisch Spectrum Twente, Enschede, NLD

**Keywords:** biomarkers, pneumonia, mr-proadm, mr-proanp, apache iv, sofa

## Abstract

Background

We assessed the ability of baseline and serial measurements of mid-regional proadrenomedullin (MR-proADM) and mid-regional proatrial natriuretic peptide (MR-proANP) to predict 28-day mortality in critically ill patients with pneumonia compared with Acute Physiological and Chronic Health Evaluation IV (APACHE IV) model and Sequential Organ Failure Assessment (SOFA) score.

Methodology

Biomarkers were collected for the first five days in this retrospective observational cohort study. Biomarker clearance (as a percentage) was presented as biomarker decline in five days. We investigated the relationship between biomarkers and mortality in a multivariable Cox regression model. APACHE IV and SOFA were calculated after 24 hours from intensive care unit admission.

Results

In 153 critically ill patients with pneumonia, 28-day mortality was 26.8%. Values of baseline MR-proADM, MR-proANP, and APACHE IV were significantly higher in 28-day nonsurvivors, but not significantly different for SOFA score. Baseline MR-proADM and MR-proANP, APACHE IV, and SOFA had a low area under the curve in receiver operating characteristics (ROC) curves. No optimal cut-off points could be calculated. Biomarkers and severity scores were divided into tertiles. The highest tertiles baseline MR-proADM and MR-proANP were not significant predictors for 28-day mortality in a multivariable model with age and APACHE IV. SOFA was not a significant predictor in univariable analysis. Clearances of MR-proADM and MR-proANP were significantly higher in 28-day survivors. MR-proADM and MR-proANP clearances had similar low accuracy to identify nonsurvivors in ROC curves and were divided into tertiles. Low clearances of MR-proADM and MR-proANP (first tertiles) were significant predictors for 28-day mortality (hazard ratio [HR]: 2.38; 95% confidence interval [CI]: 1.21-4.70; p = 0.013 and HR: 2.27; 95% CI: 1.16-4.46; p = 0.017) in a model with age and APACHE IV.

Conclusions

MR-proADM and MR-proANP clearance performed better in predicting 28-day mortality in a model with age and APACHE IV compared with single baseline measurements in a mixed population of critically ill with pneumonia.

## Introduction

Pneumonia is an important reason for intensive care unit (ICU) admission, length of stay (LOS), and death [1-3]. Mortality rates for community-acquired pneumonia (CAP) admitted to the ICU of 20-30% are reported [4]. For hospital-acquired pneumonia (HAP), mortality rates may be as high as 30 to 70% [5], and attributable mortality of 33 to 50% in patients with ventilator-associated pneumonia (VAP) has been reported [5]. Knowledge of prognostic factors predicting outcome in pneumonia may help grade its severity and predict treatment response.

The Acute Physiological and Chronic Health Evaluation IV (APACHE IV) model and the Sequential Organ Failure Assessment (SOFA) score were developed to assess disease severity or severity of organ dysfunction and predict outcome in critically ill patients [6]; however, their complexity hampers their incorporation in daily routine [6]. Additionally, biomarkers have been proposed as surrogates for these clinical scores to predict outcomes. Conventional biomarkers such as white blood count (WBC), C-reactive protein (CRP), procalcitonin (PCT), and lactate have low prognostic value in predicting mortality in patients with CAP or sepsis [7-9]. There may be a role for new cardiovascular biomarkers, namely, mid-regional proadrenomedullin (MR-proADM) and mid-regional proartrial natriuretic peptide (MR-proANP), to predict mortality in critically ill patients with pneumonia. We investigated the role of the precursor fragments of the prohormones of adrenomedullin (ADM) and atrial natriuretic peptide (ANP) because they are more stable than their respective hormones [10,11], making these assays more feasible for clinical purposes. MR-proADM is the mid-region part of the prohormone of ADM, a peptide released by multiple tissues with the anti-inflammatory and antiapoptotic effects on vascular endothelial cells, protecting the microcirculation against endothelial permeability in sepsis [12]. Moreover, ADM enhances cardiac output [9]. MR-proADM levels are rapidly induced in lower respiratory tract infections [7,9,13]. Baseline MR-proADM measurements are proven to be a good predictor of both short and long-term survival in CAP patients admitted to the emergency room or ICU [7,9]. Clearance in serial MR-proADM levels of 30% or more within five days in septic patients admitted to the ICU was associated with better outcomes [14]. MR-proANP is the mid-region part of the prohormone of ANP, a hormone predominantly produced in the atrium of the heart. ANP antagonizes the renin-angiotensin-aldosterone system in response to hypertension and water and salt retention [15]. Increased levels of MR-proANP have been reported in nonsurvivors of patients with CAP, VAP, and sepsis [15-17].

In the present study, we aimed to investigate the prognostic value of MR-proADM and MR-proANP at baseline compared with the APACHE IV model and SOFA score to predict 28-day mortality in a cohort of critically ill patients with pneumonia of any cause. Our secondary aim was to predict 28-day mortality by clearance of MR-proADM and MR-proANP using serial measurements during five days in comparison with the APACHE IV model and SOFA score. Preliminary results of this study were previously presented as a meeting abstract at the Society of Critical Care Medicine Annual Scientific Meeting on February 26, 2018.

## Materials and methods

Study design and selection criteria

This observational cohort study is a post hoc analysis of the Stop Antibiotics on Procalcitonin Guidance Study (SAPS) [18], a randomized controlled trial, in which the efficacy and safety of PCT guidance in reducing the duration of antibiotic treatment in critically ill patients in 15 Dutch ICUs were investigated from 2009 until 2013. Clinical data and microbiological and laboratory results were prospectively collected during this period. The study protocol was approved by the ethics committee of the VU University Medical Centre (Amsterdam, Netherlands). Informed consent was obtained from all participating patients of SAPS, and all patients agreed that blood samples were stored for further research. Eligible patients were critically ill patients with pneumonia from one of the participating centers of the SAPS trial (Elisabeth-TweeSteden hospital). MR-proADM and MR-proANP were measured in samples of these patients. Exclusion criteria include age <18 years, no diagnosis of pneumonia, incomplete data to calculate APACHE IV and SOFA at baseline, and unavailability of plasma samples on admission. Pneumonia was characterized by a new or progressive and persistent infiltrate on chest imaging together with fever, leukocytosis, leukopenia, or altered mental status, and at least one of the following: new onset of purulent sputum, new-onset cough, rales or bronchial breath sound, or worsening gas exchange [19]. CAP is defined as pneumonia acquired outside a hospital or a long-term care facility [1-3]. HAP refers to pneumonia that occurs 48 hours or more after admission [5]. VAP is defined as pneumonia that arises more than 48-72 hours after endotracheal intubation [5]. We followed the Strengthening the Reporting of Observational Studies in Epidemiology Statement guidelines for reporting observational studies [20].

Procedures

Blood samples were collected in EDTA tubes on a daily basis during the SAPS trial. Plasma was separated by centrifugation and stored in aliquots at -80°C. MR-proADM and MR-proANP concentrations were retrospectively measured using an automated immunofluorescent sandwich assay on a Kryptor Compact Plus analyzer (Brahms AG, Henningsdorf, Germany) at the clinical laboratory in Enschede, Netherlands. The Kryptor measures the signal emitted from an immunocomplex by time-resolved amplified cryptate emission. MR-proADM and MR-proANP assay have a limit of detection of 0.05 nmol/L and 2.1 pmol/L and functional sensitivity (lowest value with an interassay coefficient of variation [CV] <20%, as described by the manufacturer) of 0.23 nmol/L (MR-proADM) and 4.5 pmol/L (MR-proANP), respectively. The imprecision of both assays was verified according to the Clinical & Laboratory Standards Institute Evaluation Protocol 15 (CLSI EP15), using a low and high sample, measured for five days in triplicate. Between- and within-run CVs were all below 5%. APACHE IV and SOFA were extracted from the SAPS database.

Statistical analysis

All non-normally distributed data (Kolmogorov-Smirnov test; p < 0.05) were expressed as median (with interquartile range, IQR) or as the number of patients (percentage) where appropriate. Patient characteristics and outcomes were compared using a Mann-Whitney U-test for skewed distributed continuous variables and a chi-square test for categorical variables. Clearances of MR-proADM and MR-proANP were calculated by dividing the decline in biomarkers from day one to day five by the value of day one and were presented as percentages. The association between mortality and each biomarker and clinical score at admission and clearances of biomarkers was assessed using the area under the receiver operating characteristics (ROC) curves. Biomarkers and clinical scores at admission and biomarker clearance were separated in tertiles. Univariable and multivariable Cox proportional hazards regression analyses were done to study the effects on the outcome. Potential confounding variables were selected based on univariable regression analysis (variables that yielded p < 0.05) and were subsequently included in the multivariable regression analysis. The model was checked for intercorrelations among the predictor variables by collinearity statistics. All tests were two-sided and a p-value of <0.05 was considered statistically significant. All data were analyzed using the Statistical Package for the Social Sciences version 24 (IBM Corp., Armonk, NY, USA).

## Results

Descriptive characteristics of the patients

**Figure 1:**
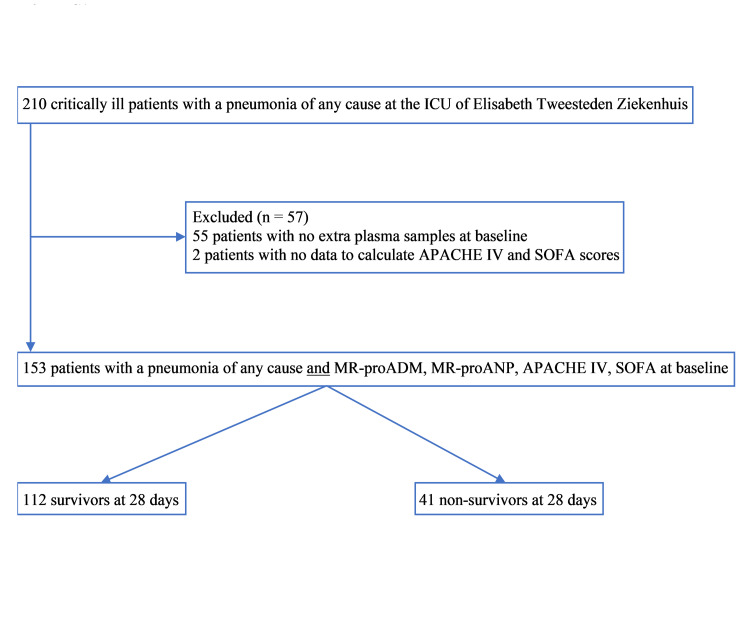
Patient flow diagram. ICU: intensive care unit; APACHE IV: Acute Physiology and Chronic Health Evaluation IV; SOFA: Sequential Organ Failure Assessment; MR-proADM: mid-regional proadrenomedullin; MR-proANP: mid-regional proatrial natriuretic peptide

We selected a cohort of 210 patients with pneumonia from the original SAPS database. In 153 patients, MR-proADM and MR-proANP concentrations were measured at baseline in addition to APACHE IV and SOFA scores. The patient flow diagram shows the flow of patients along with the primary endpoint of 28-day survival (Figure 1).

Demographics and clinical characteristics of these 153 patients, including MR-proADM, MR-proANP, APACHE IV, and SOFA score at baseline are shown in Table 1. The 28-day all-cause mortality was 26.8%. Patients were divided into survivors and nonsurvivors with regards to survival for up to 28 days. Both groups were comparable except for older age and higher APACHE IV in nonsurvivors. There was no significant difference in SOFA score on the first day between both groups. Bacterial pathogens could be detected in 64 patients (41.8%). Gram-positive microorganisms were found in 18 patients (*Streptococcus pneumoniae* n = 14, *Enterococcus spp*. n = 2, others n = 2). Gram-negative microorganisms were found in 46 patients (*Haemophilus influenzae* n = 8,* *Escherichia coli n = 8, *Klebsiella spp.* n = 8, *Pseudomonas aeruginosa* n = 6, *Enterobacter spp.* n = 4, *Haemophilus parainfluenza* n = 2, *Moraxella catarrhalis *n = 2, others n = 8). The database revealed no data on viral or fungal infections.

**Table 1:** Clinical characteristics of patients at baseline with regards to survival up to 28 days. All continuous data are presented as median (interquartile range) and categorical data as number (percentage). COPD: chronic obstructive pulmonary disease; APACHE IV: Acute Physiology and Chronic Health Evaluation IV; SOFA: Sequential Organ Failure Assessment; ICU LOS: length of stay at the intensive care; WBC: white blood cell; CRP: C-reactive protein; PCT: procalcitonin; MR-proADM: mid-regional proadrenomedullin; MR-proANP: mid-regional proatrial natriuretic peptide

	Total	Survivors	Nonsurvivors	P-value
	(N = 153)	(N = 112)	(N = 41)	
Age (years) (median, IQR)	65 (53-74)	63 (51-71)	69 (63-78)	0.003
Male gender (N, %)	94 (61.4%)	66 (58.9%)	28 (68.3%)	0.292
Patient category (N, %)				
Medical	103 (67.3%)	71 (63.4%)	32 (78%)	0.090
Surgical	27 (17.6%)	20 (17.9%)	7(17.1%)	
Trauma	23 (15%)	21 (18.8%)	2 (4.9%)	
Preexisting comorbidities (N, %)				
Congestive heart failure	15 (9.8%)	8 (7.1%)	7 (17.1%)	0.067
COPD	40 (26.1%)	25 (22.3%)	15 (36.6%)	0.075
Diabetes mellitus	26 (17%)	17 (15.2%)	9 (22%)	0.323
Cerebrovascular disease	37 (24.2%)	23 (20.5%)	14 (34.1%)	0.082
Malignancy	21 (13.7%)	14 (12.5%)	7 (17.1%)	0.467
Chronic renal disease	9 (5.9%)	5 (4.5%)	4 (9.8%)	0.218
Type of pneumonia (N, %)				
Community-acquired pneumonia	60 (39.2%)	43 (38.4%)	17 (41.5%)	0.917
Hospital-acquired pneumonia	76 (49.7%)	56 (50%)	20 (48.8%)	
Ventilator-associated pneumonia	17 (11.1%)	13 (11.6%)	4 (9.8%)	
Microbiology				
Gram-positive (N, %)	18 (11.8%)	14 (12.5%)	4 (9.8%)	0.151
Gram-negative (N, %)	46 (30%)	38 (33.9%)	8 (19.5%)	
No bacterial pathogens (N, %)	89 (58.2%)	60 (53.6%)	29 (70.7%)	
Severity of illness				
Sepsis-3, sepsis (N, %)	148 (96.7%)	109 (97.3%)	39 (95.1%)	0.498
Sepsis-3, septic shock (N, %)	27 (17.8%)	18 (16.1%)	9 (22.5%)	0.361
APACHE IV (points) (median, IQR)	73 (53 - 86)	65 (50 - 84)	81 (61 - 100)	0.003
SOFA score (points) (median, IQR)	5 (3 – 8)	5 (3 - 7)	6 (3 - 10)	0.441
Treatment upon diagnosis (N, %)				
Mechanical ventilation	125 (81.7%)	90 (80.4%)	35 (85.4%)	0.478
Vasopressor use	144 (94.1%)	105 (93,8%)	39 (95.1%)	0.749
Renal replacement therapy	7 (4.6%)	5 (4.5%)	2 (4.9%)	0.914
Length of stay				
ICU LOS (days) (median, IQR)	9 (5-18)	9 (4-22)	9 (5-15)	0.754
Biomarkers				
WBC, 10^9^/L, (median, IQR)	12.6 (9.3-17.6)	12.3 (9.3-16.5)	13.5 (9.5-19)	0.407
CRP (mg/L), (median, IQR)	142 (81.8-251)	158 (82-267)	111 (66-230)	0.161
PCT (ng/mL), (median, IQR)	0.7 (0.2-4.3)	0.6 (0.2-3.1)	1.3 (0.3-12.2)	0.066
Lactate (mmol/L), (median, IQR)	1.3 (1-1.8)	1.3 (1-1.8)	1.5 (1-2.1)	0.147
MR-proADM (nmol/L), (median, IQR)	1.3 (0.9-2.5)	1.3 (0.9-2.1)	1.8 (1-3.3)	0.017
MR-proANP (pmol/L), (median, IQR)	159.8 (87.7-313)	141.7 (79.1-208)	201.9 (147.1-454.3)	0.001

Association between biomarkers at baseline and 28-day mortality

**Figure 2:**
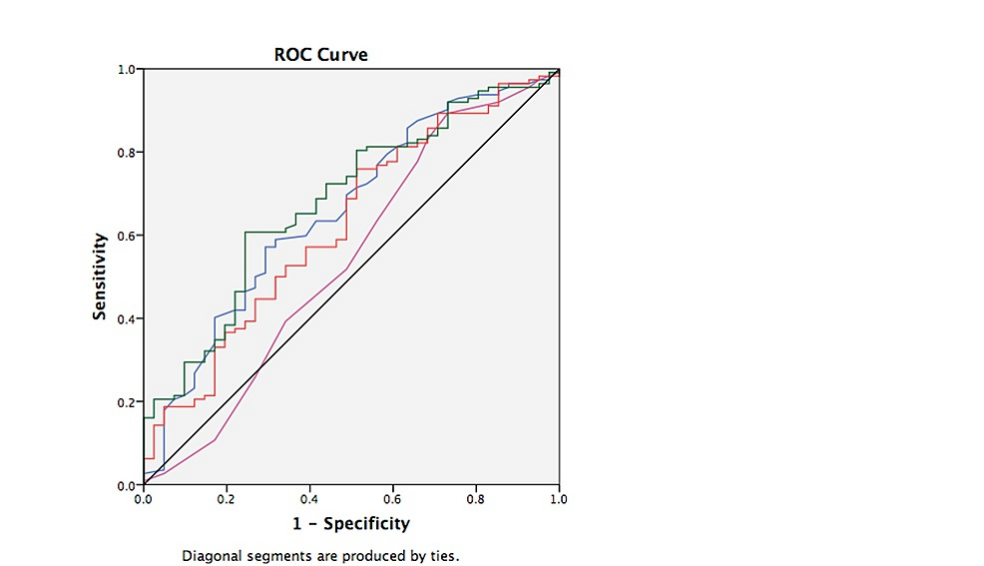
Receiver operating characteristics curve for biomarkers/clinical scores on day one in predicting 28-day mortality. Red line = MR-proADM (AUC: 0.63; 95% CI: 0.53-0.73; p = 0.017), green line = MR-proANP (AUC: 0.68; 95% CI: 0.59-0.78; p = 0.001), blue line = APACHE IV (AUC: 0.66; 95% CI: -0.56-0.76;p = 0.003), violet line = SOFA (AUC: 0.54; 95% CI: 0.43-0.65; p = 0.444), black line = reference line. MR-proADM: mid-regional proadrenomedullin; MR-proANP: mid-regional proatrial natriuretic peptide; APACHE IV: Acute Physiology and Chronic Health Evaluation IV; SOFA: Sequential Organ Failure Assessment

Nonsurvivors at 28 days had significantly higher concentrations of MR-proADM and MR-proANP at baseline than survivors (Table 1). WBC, CRP, PCT, and lactate concentrations were not significantly different. The area under the curve (AUC) in ROC analysis for the prediction of 28-day mortality of baseline MR-proADM (0.63; 95% confidence interval (CI): 0.53-0.73; p = 0.017)) and MR-proANP (0.68; 95% CI: 0.59-0.78; p = 0.001)) was low (Table 2). AUCs for APACHE IV and SOFA were 0.66 (95% CI: 0.56-0.76; p = 0.003) and 0.54 (95% CI: 0.43-0.65; p = 0.444), respectively (Figure 2) (Table 2). Combinations of biomarkers and scores did not yield a higher AUC (Table 2). Due to a low AUC in the ROC analysis, no optimal cut-off points could be calculated. MR-proADM, MR-proANP, APACHE IV, and SOFA were divided into tertiles. The highest tertile of MR-proADM, MR-proANP, and APACHE IV on day 1 had the strongest association in predicting 28-day mortality compared to patients with the two lower tertiles in univariable Cox regression analysis (Table 3). SOFA score was not a significant predictor of 28-day mortality in univariable analysis and was excluded from the multivariable analysis. The highest tertiles baseline MR-proADM and MR-proANP were not significant predictors for 28-day mortality in a multivariable model with age and APACHE IV. Only APACHE IV had a significant contribution (hazard ratio [HR]: 2.07, 95% CI: 1.11-3.86; p =0.021) (Table 3). There were no signs of high correlations between the predictor variables in the model. Tolerance values were between 0.75 and 0.98 (Table 4).

**Table 2:** Prediction of 28-day mortality by biomarkers and clinical scores on the first day. Association between biomarkers and clinical scores with 28-day mortality by receiver operating characteristics curves. AUC: area under the curve; 95% CI: 95% confidence interval; MR-proADM: mid-regional proadrenomedullin; MR-proANP: mid-regional proartrial natriuretic peptide; APACHE IV: Acute Physiological and Chronic Health Evaluation IV; SOFA: Sequential Organ Failure Assessment

Biomarker or clinical score	Patients (N)	Mortality (N)	AUC (95% CI)	P-value
MR-proADM	153	41	0.63 (0.53-0.73)	0.017
MR-proANP	153	41	0.68 (0.59-0.78)	0.001
APACHE IV	153	41	0.66 (0.56-0.76)	0.003
SOFA	153	41	0.54 (0.43-0.65)	0.444
APACHE IV + MR-proADM	153	41	0.68 (0.58-0.78)	0.001
APACHE IV + MR-proANP	153	41	0.69 ((0.59-0.79)	<0.001
SOFA + MR-proADM	153	41	0.61 (0.50-0.72)	0.039
SOFA + MR-proANP	153	41	0.68 (0.59-0.78)	0.001
MR-proADM + MR-proANP	153	41	0.68 (0.59-0.77)	0.001

**Table 3:** Univariable and multivariable Cox regression models for the prediction of 28-day mortality with baseline biomarker values. HR: hazard ratio; CI: confidence interval; SOFA: Sequential Organ Failure Assessment; APACHE IV: Acute Physiology and Chronic Health Evaluation IV; MR-proADM: mid-regional proadrenomedullin; MR-proANP: mid-regional proatrial natriuretic peptide

	Univariable analysis		Multivariable analysis	
	HR (95% CI)	P-value	HR (95% CI)	P-value
Age	1.03 (1.01-1.06)	0.014	1.02 (0.99-1.05)	0.157
SOFA				
Low (1^st^ + 2^nd^ tertiles)	1.0 (Reference)		-	-
High (3^rd^ tertile)	1.25 (0.68-2.32)	0.476	-	-
APACHE IV				
Low (1^st^ + 2^nd^ tertiles)	1.0 (Reference)		1.0 (Reference)	
High (3^rd^ tertile)	2.22 (1.20-4.10)	0.011	2.07 (1.11-3.86)	0.021
MR-proADM day 1				
Low (1^st^ + 2^nd^ tertiles)	1.0 (Reference)		1.0 (Reference)	
High (3^rd^ tertile)	2.12 (1.15-3.91)	0.017	1.09 (0.50-2.38)	0.823
MR-proANP day 1				
Low (1^st^ + 2^nd^ tertiles)	1.0 (Reference)		1.0 (Reference)	
High (3^rd^ tertile)	2.42 (1.31-4.47)	0.005	1.87 (0.91-3.83)	0.089

**Table 4:** Collinearity statistics of biomarkers/clinical scores on the first day in multivariable Cox regression model.

	Tolerance value
Age	0.79
APACHE IV	0.98
MR-proADM	0.69
MR-proANP	0.75

Association between biomarker kinetics and 28-day mortality

**Figure 3:**
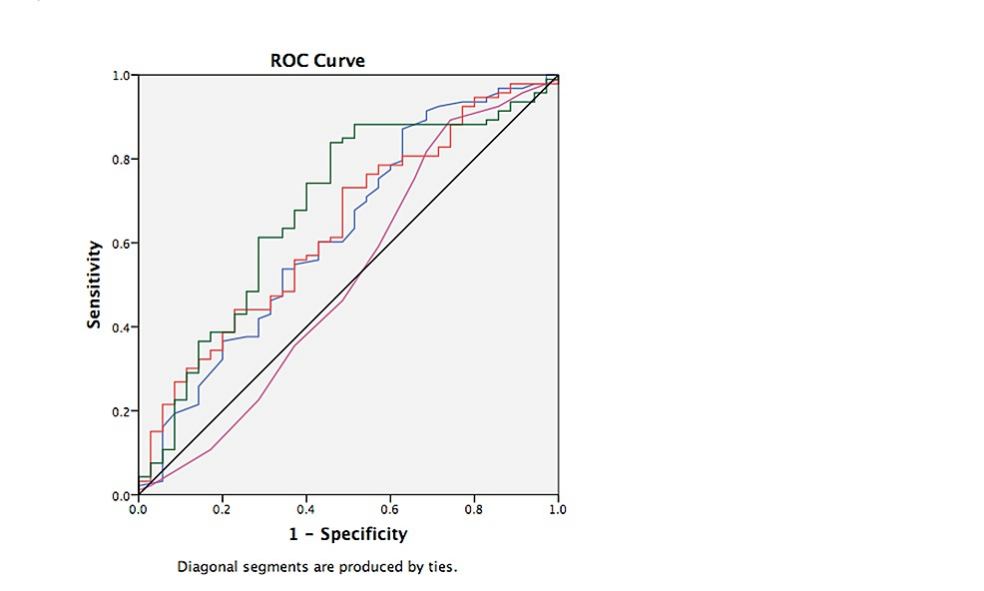
Receiver operating characteristics curve biomarker clearance in five days in predicting 28-day mortality. Red line = MR-proADM clearance (AUC: 0.66; 95% CI: 0.56-0.76; p= 0.004), green line = MR-proANP clearance  (AUC: 0.68; 95% CI: 0.57-0.79; p = 0.001), blue line = APACHE IV (AUC: 0.66; 95% CI: -0.56-0.76; p = 0.003), violet line = SOFA (AUC: 0.54, 95% CI: 0.43-0.65;p = 0.444), black line = reference line. MR-proADM: mid-regional proadrenomedullin; MR-proANP: mid-regional proatrial natriuretic peptide; APACHE IV: Acute Physiology and Chronic Health Evaluation IV; SOFA: Sequential Organ Failure Assessment

Clearance percentages in five days of MR-proADM and MR-proANP were calculated and compared between survivors and nonsurvivors in 28 days. Clearances of MR-proADM and MR-proANP were significantly higher in survivors compared to nonsurvivors in 28 days (Table 5). MR-proADM and MR-proANP clearance had low accuracy to identify nonsurvivors in ROC curves with AUC of 0.66 (95% CI: 0.56-0.76;p = 0.004) and 0.68 (95% CI: 0.257-0.78; p = 0.002), respectively (Figure 3) (Table 6). A combination of biomarker clearance did not yield a substantially higher AUC (Table 6). Clearances of MR-proADM and MR-proANP were divided into tertiles. Univariable Cox regression analysis demonstrated that the lowest MR-proADM and MR-proANP clearance (first tertile) in five days had a strong association in predicting 28-day mortality compared to patients with the combined second and third tertiles (Table 7). SOFA score was excluded from further multivariable analysis. Low clearances of MR-proADM and MR-proANP (first tertiles) were significant predictors for 28-day mortality (HR: 2.38; 95% CI: 1.21-4.70; p = 0.013 and HR: 2.27; 95% CI: 1.16-4.46; p = 0.017, respectively) in a model with age and APACHE IV (Table 7). There were no signs of high correlations between the predictor variables, and tolerance values were between 0.94 and 0.98 (Table 8).

**Table 5:** MR-proADM and MR-proANP clearance in five days. All continuous data are presented as median percentage (interquartile range) MR-proADM: mid-regional proadrenomedullin; MR-proANP: mid-regional proatrial natriuretic peptide

28-day survival	Survivors	Nonsurvivors	P-value
	N = 112	N = 41	
MR-proADM clearance (%)	34% (16-50)	16% (-6-37)	0.004
MR-proANP clearance (%)	14% (0-34)	-9% (-28-18)	0.002

**Table 6:** Prediction of 28-day mortality by biomarker clearance in five days. Association between biomarker clearance with 28-day mortality by receiver operating characteristics curves AUC: area under the curve, CI: confidence interval; MR-proADM: mid-regional proadrenomedullin; MR-proANP: mid-regional proatrial natriuretic peptide; APACHE IV: Acute Physiology and Chronic Health Evaluation IV; SOFA: Sequential Organ Failure Assessment

Biomarker	Patients (N)	Mortality (N)	AUC (95% CI)	P-value
MR-proADM clearance	137	38	0.66 (0.56-0.76)	0.004
MR-proANP clearance	130	35	0.68 (0.57-0.78)	0.002
APACHE IV + MR-proADM clearance	137	38	0.70 (0.60-0.80)	<0.001
APACHE IV + MR-proANP clearance	130	35	0.71 (0.62-0.81)	<0.001
SOFA + MR-proADM clearance	137	38	0.68 (0.58-0.77)	0.002
SOFA + MR-proANP clearance	130	35	0.69 (0.58-0.79)	0.001
MR-proADM clearance + MR-proANP clearance	128	35	0.69 (0.59-0.79)	0.001

**Table 7:** Univariable and multivariable Cox regression models for the prediction of 28-day mortality with biomarker clearance in five days. HR: hazard ratio; CI: confidence interval; SOFA: Sequential Organ Failure Assessment; APACHE IV: Acute Physiology and Chronic Health Evaluation IV; MR-proADM: mid-regional proadrenomedullin; MR-proANP: mid-regional proatrial natriuretic peptide

	Univariable analysis		Multivariable analysis	
	HR (95% CI)	P-value	HR (95% CI)	P-value
Age	1.03 (1.01-1.06)	0.014	1.03 (1.01-1.06)	0.033
SOFA				
Low (1^st^ + 2^nd^ tertiles)	1.0 (Reference)		-	-
High (3^rd^ tertile)	1.25 (0.68-2.32)	0.476	-	-
APACHE IV				
Low (1^st^ + 2^nd^ tertiles)	1.0 (Reference)		1.0 (Reference)	
High (3^rd^ tertile)	2.22 (1.20-4.10)	0.011	1.71 (0.87-3.37)	0.122
MR-proADM clearance				
2^nd^ + 3^rd^ tertiles (≥17% drop)	1.0 (Reference)		1.0 (Reference)	
1^st^ tertile (<17% drop)	2.52 (1.33-4.76)	0.004	2.38 (1.21-4.70)	0.013
MR-proANP clearance				
2^nd^ + 3^rd^ tertiles (≥0% drop)	1.0 (Reference)		1.0 (Reference)	
1^st^ tertile (<0% drop)	2.80 (1.44-5.45)	0.002	2.27 (1.16-4.46)	0.017

**Table 8:** Collinearity statistics of biomarker clearance in five days in multivariable Cox regression model. APACHE IV: Acute Physiology and Chronic Health Evaluation IV; MR-proADM: mid-regional proadrenomedullin; MR-proANP: mid-regional proatrial natriuretic peptide

	Tolerance value
Age	0.94
APACHE IV	0.94
MR-proADM clearance	0.98
MR-proANP clearance	0.98

## Discussion

The primary aim of our study was to investigate the prognostic value of baseline MR-proADM and MR-proANP compared with APACHE IV and SOFA scores to predict 28-day mortality in a well-described cohort of critically ill patients with pneumonia. The secondary aim was the prediction of 28-day mortality by clearances of MR-proADM and MR-proANP in five days compared with both severity scores. We reported two main findings. First, single baseline MR-proADM and MR-proANP levels were not superior to APACHE IV in both ROC curves and in a multivariable Cox regression model for the prediction of 28-day mortality. SOFA score performed poorly in identifying nonsurvivors. Second, MR-proADM and MR-proANP clearance in five days performed better in predicting 28-day mortality in a model with age and APACHE IV. However, APACHE IV and SOFA scores require time and effort to collect data and are only available after 24 hours from admission [6]. A single baseline biomarker, with the ability to provide prognostic information early after admission, would have an advantage. Unfortunately, single baseline MR-proADM and MR-proANP did not perform well in our study. The addition of a single baseline MR-proADM and MR-proANP to the APACHE IV and SOFA models did not increase their predictive value. Clearances of biomarkers MR-proADM and MR-proANP performed better in the Cox regression model but required five days to calculate in our study.

Our findings of a limited value of baseline MR-proADM and MR-proANP in predicting short-term survival in critically ill patients with pneumonia are in contrast with several studies. These two biomarkers have been studied frequently in patients with respiratory infections [7,9,13,16,17]. However, most patients were not admitted to the ICU [7,9,13,16]. Baseline MR-proADM and MR-proANP were studied in an observational cohort study of 728 CAP patients admitted to the emergency department (ED) [9]. Only 18 patients were admitted to the ICU. MR-proADM had the best performance for the prediction of 28-day mortality (HR: 3.67 and AUC: 0.85). MR-proADM proved to have the highest HR in predicting 28-day mortality in an observational cohort study of 1,175 ED patients [21]. Only 32 patients were admitted to the ICU. Baseline MR-proADM performed well in comparison with APACHE II and Simplified Acute Physiology Score II (SAPS II) in another observational study [22]. Pneumonia patients were not selected in this study as all patients admitted to the ICU were included. Our findings of better performance of MR-proADM clearance in five days as a prognostic factor in predicting mortality were supported by a study in which clearance of MR-proADM of 30% or more in five days had a higher survival probability in 100 days among septic ICU patients [14].

MR-proADM and MR-proANP are predominantly cardiovascular biomarkers. A possible reason for the elevation of cardiovascular biomarkers in acute pulmonary disease may be transient pulmonary hypertension, resulting in right heart strain [7,9]. An explanation for the observed lower prognostic value of the biomarkers in our cohort could be underlying cardiac failure [23]. The database was searched for comorbidities, and only 15 patients had congestive heart failure as comorbidity, but there were no significant differences between both groups. Septic cardiomyopathy could be another reason for elevated biomarkers, and almost all of our patients were septic according to Sepsis-3 definitions, but well balanced between survivors and nonsurvivors. Renal failure is another reason for the elevation of MR-proADM and MR-proANP, most probably due to inappropriate renal clearance or increased strain on the atria due to fluid overload [9,24]. A small number of patients had chronic renal disease as comorbidity, and a small portion of the cohort was on renal replacement therapy the first day. As day one biomarkers had low prognostic accuracy regarding survival, biomarkers may need time to differentiate between survival and death. Indeed, MR-proADM and MR-proANP clearance in five days performed better in predicting mortality. Persistent high levels of biomarkers in nonsurvivors may be due to persistent synthesis and release due to active organ dysfunction [14]. This hypothesis is supported by a prospective cohort study with 114 septic critically ill patients with cancer [25]. MR-proADM levels decreased from baseline to follow-up (four to seven days later) in survivors and increased in nonsurviving patients in this study. Moreover, MR-proANP levels increased in five days in nonsurvivors, resulting in a negative calculated clearance percentage of MR-proANP in our cohort. Persistently increased levels of MR-proANP in 10 days following VAP onset were also reported in nonsurviving patients in another study [17].

Some limitations of our study need to be addressed. First, we performed a retrospective observational study in a cohort of critically ill patients with pneumonia selected from the SAPS database. We must rely on older data of clinical practice leading to potential observational bias. Second, by selecting 153 patients out of 210 potential eligible critically ill patients with pneumonia and index tests, we introduced selection bias. Third, our study population is relatively small and heterogeneous in source as patients with CAP, HAP, and VAP were included. These subgroups were too small to analyze by diagnostic subgroup. Both observational and selection bias may have led to potential underestimation of the prognostic performance of MR-proADM and MR-proANP, and we, therefore, plan to conduct a larger prospective observational study in the near future.

A strong feature of our analysis is that it is a real-life study performed in a mixed surgical and medical ICU with patients with pneumonia consisting of CAP, HAP, and VAP, reflecting routine clinical ICU practice.

## Conclusions

Single baseline MR-proADM and MR-proANP were not superior to APACHE IV in predicting 28-day mortality in a mixed population of critically ill patients with pneumonia. SOFA score was not a significant predictor of 28-day mortality. The predictive value of serial-measured MR-proADM and MR-proANP in predicting 28-day mortality in a model with age and APACHE IV exceeded those of single baseline MR-proADM and MR-proANP measurements in this population. Therefore, clearance of MR-proADM and MR-proANP in time may be better used for prognostication instead of single values.
